# The global neurosurgical postcode lottery: Geographic and socioeconomic drivers of unequal treatment worldwide on behalf of diversity in neurosurgery committee and ethico-legal committee of the EANS

**DOI:** 10.1016/j.bas.2026.106143

**Published:** 2026-06-23

**Authors:** Mirko Micovic, Tijana Ilic, Mario Ganau, Andrew F. Alalade, Bojana Zivkovic

**Affiliations:** a Neurosurgery Clinic, University Clinical Center of Serbia, Dr Koste Todorovica 4, Belgrade, 11000, Serbia; b Faculty of Medicine, University of Belgrade, Dr Subotica 8, Belgrade, 11000, Serbia; c Institute of Spinal Column and Neurosciences-Clinique Bizet, 23 Rue Georges Bizet, Paris, 75116, France; d Department of Neurosurgery, John Radcliffe Hospital, Oxford University Hospitals NHS Foundation Trust, Headley Way, Headington, Oxford, Oxfordshire, OX3 9DU, United Kingdom; e School of Medicine, BAU International University Batumi, 6010 Batumi, Georgia; f University of Lancashire, Fylde Road, Lancashire, Preston, PR1 2HE, United Kingdom; g Department of Neurosurgery, Royal Preston Hospital, Lancashire Teaching Hospitals NHS Foundation Trust, Sharoe Green Lane North, Fulwood, Lancashire, Preston, PR2 9HT, United Kingdom

**Keywords:** Global neurosurgery, Health equity, Geographic disparity, Socioeconomic determinants, Brain drain

## Abstract

• The neurosurgical postcode lottery reflects global inequity.
• Geography and income strongly shape access to neurosurgical care worldwide.
• Workforce shortages leave millions without access to lifesaving neurosurgery.
• Costs, travel distance, and discrimination deepen treatment inequality.
• Equity requires workforce growth, stronger health systems, and fair financing.

## 1 Introduction: two women, two worlds

The Caribbean sun hung low when Margaret, a 63-year-old British tourist, was struck without warning by an excruciating pain that drove her to the floor. A ruptured cerebral aneurysm. Within minutes, a bystander had retrieved her health insurance card and dialled the emergency number on the back. A medical jet broke through the clouds. Roughly an hour after collapse, Margaret was on the operating table of an elite surgical centre, where an experienced surgeon clipped with precision to the lesion deep within her brain. She regained consciousness two weeks later. Within a year, the ordeal had receded to something like a distant nightmare.

A world away, on a blazing Sunday in rural southeastern Europe, Lydia, a 44-year-old farmworker, noticed a mild headache since morning. She had lived with hypertension for years but disliked the small red pills. As dusk fell, she continued working alone in the fields. Suddenly, everything went dark. Her husband found her after midnight and loaded her into a neighbour's worn car, driving toward the nearest medical outpost, where a drowsy doctor stamped a referral and sent them on with an Ambu bag and little else. A flat tyre slowed them further. By the time Lydia reached the city hospital around dawn, the angiography unit had been out of service since Friday. Surgery without adequate imaging, with a severe shortage of clips and materials, was no option at all. Lydia survived until Wednesday. A week later, a handful of friends gathered around her burial mound.

Margaret lived because of her geographical and economic circumstances. Lydia died for precisely the same reasons. The disparity between them reflects not a difference in skill or clinical possibility, but a global health system that has allowed medicine to function as a privilege rather than a right. These two women are not outliers. Multiplied across millions of patients each year, their stories constitute what we call the global neurosurgical postcode lottery.

Neurosurgeons are trained to interpret the language of absence: the silent cortex on an electroencephalogram, the darkness of a ventricle expanded by hydrocephalus, the ominous hyperdensity of a haematoma on computed tomography. By profession, we are interpreters of the void. Yet the greatest void we confront is invisible to any scanner: the immense gap chronicled in global health data (Meara et al., 2015). While neurosurgery in wealthy regions operates in the era of microsurgery and endovascular intervention, more than five billion people remain constrained to a reality closer to Harvey Cushing's era (Chaurasia, 2023). The global neurosurgical workforce stands at fewer than one surgeon per 100,000 population, and 33 countries have no practising neurosurgeon at all (Gupta et al., 2024; Mukhopadhyay et al., 2019).

## 2 The scale of the deficit

Traumatic brain injury (TBI) alone kills an estimated 69,000 people annually in sub-Saharan Africa (Dewan et al., 2019), and this figure is almost certainly an undercount. Globally, TBI accounts for over 50 million incident cases each year. Approximately five million neurosurgical operations are left undone annually, with mortality driven by delayed transfer, absent imaging, and untreated vascular risk factors. The workforce maldistribution is extraordinary. North America and Western Europe have four to eight neurosurgeons per 100,000 population; sub-Saharan Africa averages fewer than 0.1 (Dewan et al., 2019). In the Central African Republic and South Sudan, a single surgeon serves entire nations of millions. A patient diagnosed within the first hour in a European emergency department may wait days in many Low- and Middle-Income Countries (LMIC) settings, if imaging occurs at all. By then, herniation, haemorrhage or irreversible ischaemia may have already sealed the outcome.

## 3 Geographic, structural and socioeconomic drivers

The geography of inequity is not monolithic. India has produced substantial numbers of neurosurgeons, concentrated almost entirely in metropolitan centres, while hundreds of millions in rural areas remain effectively unreached (Dewan et al., 2019). Conflict dismantles capacity rapidly: hospitals destroyed, surgeons fleeing, supply chains collapsed. The neurosurgical catastrophes of Syria, Yemen, and South Sudan show how decades of capacity-building can be reversed within months. National income is the most powerful population-level predictor of neurosurgical access. Even where services nominally exist, costs are financially catastrophic for most families: treating hydrocephalus or traumatic brain injury in Nigeria, India, or Ethiopia frequently exceeds annual household income. Gender compounds these barriers, with delayed presentation more common among women in many LMIC settings due to lower decision-making autonomy. Brain drain, the emigration of LMIC-trained neurosurgeons to high-income countries (HIC), transfers human capital whose replacement cost runs into hundreds of thousands of dollars, a structural subsidy of wealthy systems by poor ones.

The postcode lottery is not solely an international phenomenon. Within the United Kingdom, the United States, Canada, and Australia, significant geographic and socioeconomic disparities in outcomes have been well documented. In the United States, Black, Hispanic, and Native American patients experience delayed diagnosis and poorer operative outcomes even after adjustment for clinical variables, implicating structural racism (Howard et al., 2023). In the United Kingdom, socioeconomic deprivation is independently associated with lower rates of glioblastoma resection and reduced survival (Mauricaite et al., 2026).

## 4 Progress and priorities

Sustained twinning models between HIC and LMIC departments, enabling faculty development, curriculum co-design, and genuine knowledge transfer, have shown more durable benefit than episodic short-term missions, which frequently fail to build lasting capacity and carry ethical risks. Task-sharing with trained surgical officers has demonstrated comparable outcomes for burr-hole craniotomy in extreme-deficit settings. Telemedicine platforms and AI-assisted triage for haemorrhage detection have shown feasibility across LMIC contexts, though algorithmic bias in systems trained predominantly on HIC data remains a live ethical concern.

## 5 The debt we owe

The contrasting outcomes of Margaret and Lydia were not the result of differences in neurosurgical science or clinical capability, but of unequal access to care. Such inequities are neither inevitable nor acceptable. The global neurosurgical community bears an ethical responsibility to advocate for the structural changes required: workforce investment, universal health coverage, infrastructure development, and sustained political commitment. Excess morbidity and mortality in LMIC are not merely statistics. Until equitable access to essential neurosurgical care is achieved, the promise of global health equity will remain unfulfilled.

## Disclosure

The patient narratives presented at the opening of this editorial are composite fictional vignettes. They were constructed to illustrate clinical and epidemiological realities and are not based on any specific people, places, or events. Any similarities to actual individuals or circumstances are purely coincidental.

## Contributions

All authors have made substantial contributions to all of the following: (1) the conception and design of the study (M.M., B.Z.), acquisition of data, analysis and interpretation of data (T.I., M.G., A.A), (2) drafting the article and revising it (B.Z., M.G., T.I., A.A.).

## Declaration of generative AI and AI-assisted technologies

**Fig. 1:**
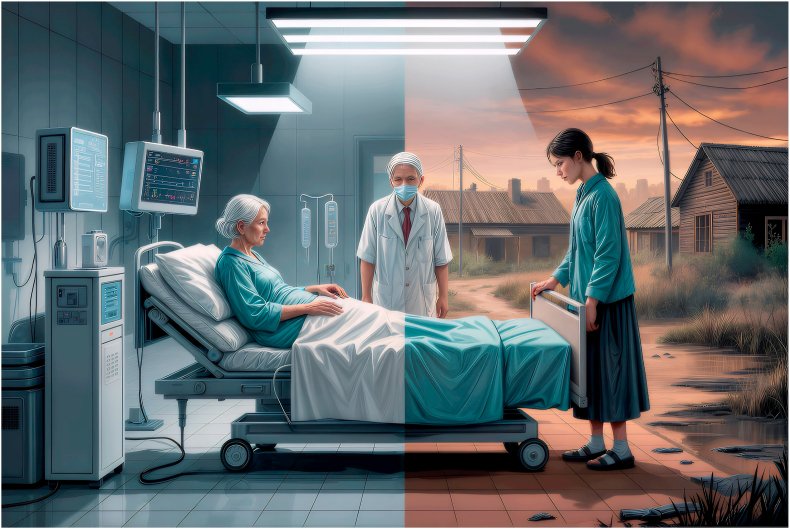
The global neurosurgical postcode lottery: Geographic and socioeconomic drivers of unequal treatment worldwide.

During the preparation of this work the author M.M. used the Bonkers Advanced Model (Merlin AI), enhanced with the KREA Upscale V1 model in order to generate Fig. 1. After using this tool, the author reviewed and edited the content as needed and takes full responsibility for the content of the figure.

## Founding sources

This research did not receive any specific grant from funding agencies in the public, commercial, or not-for-profit sectors.

## Declaration of competing interest

The authors declare that they have no known competing financial interests or personal relationships that could have appeared to influence the work reported in this paper.
